# Synthetic emmprin peptides with chitobiose substitution stimulate MMP-2 production by fibroblasts

**DOI:** 10.1186/1471-2407-11-300

**Published:** 2011-07-17

**Authors:** Takehito Kawakami, Tetsuro Sameshima, Hironobu Hojo, Kaori Koga, Yoshiaki Nakahara, Bryan P Toole, Junji Suzumiya, Yasunori Okada, Akinori Iwasaki, Kazuki Nabeshima

**Affiliations:** 1 Department of Pathology, Fukuoka University School of Medicine, 7-45-1 Nanakuma, Jonan-ku, Fukuoka 814-0180, Japan; 2 Department of Thoracic Surgery, Fukuoka University School of Medicine, 7-45-1 Nanakuma, Jonan-ku, Fukuoka 814-0180, Japan; 3 Department of Neurosurgery, NTT Medical Center Tokyo, 5-9-22 Higashigotanda, Shinagawa-ku, Tokyo 141-8625, Japan; 4 Department of Applied Biochemistry, Institute of Glycotechnology, 1117 Kitakinme, Hiratsuka, Tokai University, Kanagawa 259-1292, Japan; 5 Department of Regenerative Medicine and Cell Biology, Medical University of South Carolina, 173 Ashley Avenue, Charleston, SC 29425, USA; 6 Shimane University Hospital Cancer Center, 89-1 Enya, Izumo, Shimane 693-8501 Japan; 7 Department of Pathology, Keio University School of Medicine, 35 Shinanomachi, Tokyo 160-0016, Japan

## Abstract

**Background:**

Emmprin, a glycoprotein containing two Ig domains, is enriched on tumor cell surfaces and stimulates matrix metalloproteinase (MMP) production by adjacent stromal cells. Its first Ig domain (ECI) contains the biologically active site. The dependence of emmprin activity on N-glycosylation is controversial. We investigated whether synthetic ECI with the shortest sugar is functionally active.

**Methods:**

The whole ECI peptides carrying sugar chains, a chitobiose unit or N-linked core pentasaccharide, were synthesized by the thioester method and added to fibroblasts to examine whether they stimulate MMP-2 production.

**Results:**

ECI carrying a chitobiose unit, ECI-(GlcNAc) _2_, but not ECI without a chitobiose unit or the chitobiose unit alone, dose-dependently stimulated MMP-2 production by fibroblasts. ECI with longer chitobiose units, ECI-[(Man)_3_(GlcNAc)_2_], also stimulated MMP-2 production, but the extent of its stimulation was lower than that of ECI-(GlcNAc)_2_.

**Conclusions:**

Our results indicate that ECI can mimic emmprin activity when substituted with chitobiose, the disaccharide with which N-glycosylation starts.

## Background

Matrix metalloproteinases (MMPs) play an essential role in remodeling of extracellular matrices (ECMs) involved in various biological processes, such as inflammation, tissue regeneration and tumor invasion. Among the MMPs, gelatinase A (MMP-2) is the most abundant MMP and frequently correlates with malignant progression and invasive behavior of tumor cells [1]. *In situ *hybridization studies of human surgical specimens have shown that stromal fibroblasts are the predominant source of MMP-2 in the majority of carcinomas [2-4]. Malignant cells stimulate nearby fibroblasts to produce MMPs via soluble cytokines and growth factors or through cell surface interactions mediated by plasma membrane proteins, such as emmprin [5]. Emmprin, also known as basigin/CD147, is an integral plasma membrane glycoprotein of the Ig superfamily that contains two extracellular Ig domains [6]. Expression of emmprin is upregulated in human malignant tumors, such as breast, lung and bladder carcinomas, malignant melanomas, gliomas and lymphomas, compared with their normal counterparts [7-11]. In malignant tumors, emmprin acts as a modulator of tumor-stroma cross-talk, since it mediates not only MMP production but also tumor angiogenesis through the stimulation of vascular endothelial growth factor (VEGF) expression [12], induction of activated stromal myofibroblasts [13], and anchorage-independent growth and multidrug resistance in a hyaluronan-dependent fashion [14-16]. The activity-blocking monoclonal antibody (mAb), E11F4, recognizes the first Ig domain (ECI) of emmprin, implying that this region of emmprin contains the structure responsible for the activity of this protein [5,6]. Moreover, it is reported that emmprin-induced stimulation of MMP production in fibroblasts is dependent on N-glycosylation of its extracellular domains [17,18]. However, it has been reported recently that nonglycosylated recombinant emmprin could stimulate fibroblasts to express the mRNAs of MMP-1, 2 and 3 [19]. The objective of the present study was to determine whether the activity of synthetic emmprin ECI peptides, with or without a chitobiose unit (GlcNAc-GlcNAc), the disaccharide with which N-glycosylation starts, mimics that of emmprin. The results showed that synthetic ECI substituted with a chitobiose unit, but not ECI alone, stimulates fibroblasts to produce MMP-2.

## Methods

### Peptides

**Figure 1:**
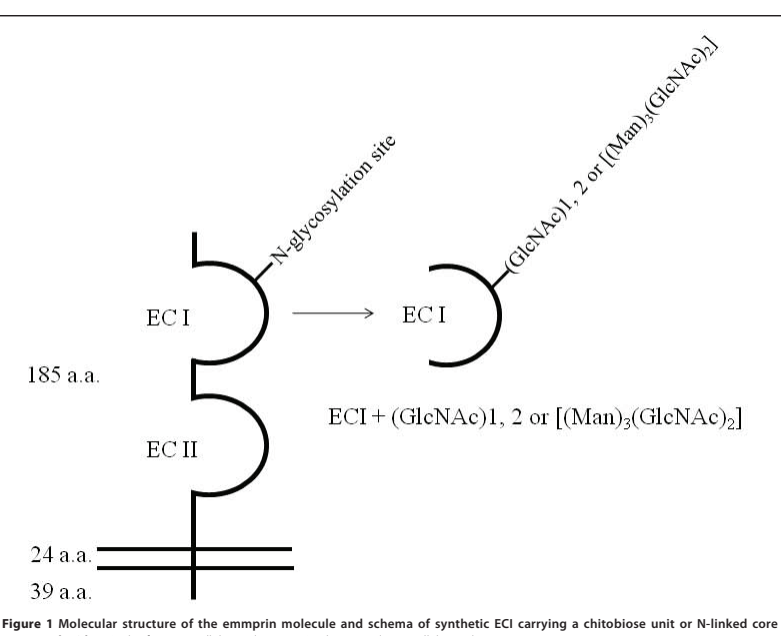
**Molecular structure of the emmprin molecule and schema of synthetic ECI carrying a chitobiose unit or N-linked core pentasaccharide**. ECI, the first extracellular Ig domain; ECII, the second extracellular Ig domain.

ECI (34-94th a.a.) carrying a chitobiose unit or N-linked core pentasaccharide at Asn^44 ^was synthesized by the thioester method as described previously (Figure 1) [20-22]. ECI carrying one or two GlcNAc or N-linked core pentasaccharide [(Man)_3_(GlcNAc)_2_] was dissolved in dimethyl sulfoxide (DMSO). The final concentration of DMSO in the culture medium was less than 0.01%. At this concentration, the solvents alone showed no cytotoxic effects or any detectable effects on MMP production.

### Cell culture

Human adult fibroblasts, MBT-3 and ST353, were obtained from a metastatic tumor in the cerebellum from a primary lung adenocarcinoma [23] and nonlesional dermis around nodular fasciitis [24], respectively. The tumor cell line FU-EPS-1 was established from a patient with epithelioid sarcoma who had not received any chemotherapy before surgical resection [25]. These cell lines were cultured in Dulbecco's modified Eagle's medium (DMEM) supplemented with 10% fetal bovine serum (FBS) and antibiotics (100 U/ml penicillin G and 100 μg/ml streptomycin) in a humidified atmosphere of 5% CO_2 _at 37°C.

### Co-culture experiments

Co-culture experiments were performed as described previously [24]. Briefly, cultures containing either fibroblasts, tumor cells or both were established in 20-mm diameter wells containing 1.0 ml DMEM with 10% FBS. For co-cultures, the same number (4 × 10^4^) of fibroblasts and tumor cells were incubated. The cells were allowed to attach for 24 h at 37°C, after which their culture media were replaced with fresh serum-free (SF) DMEM containing 0.2% lactalbumin hydrolysate (LH) (0.5 ml/well) prior to the start of the experiment. Experiments under each condition were performed in triplicate wells. The culture media were replaced with fresh SF DMEM at 3 days and harvested at 6 days. The harvested media were used for immunoblotting and enzyme immunoassay.

#### Treatment of fibroblasts with ECI-(GlcNAc)_1 or 2_, ECI-[(Man)_3_(GlcNAc)_2_], Asn44-(GlcNAc)_2_, or ECI alone

Fibroblasts were allowed to attach for 24 h in DMEM with 10% FBS, then their culture media were replaced with fresh SF DMEM containing 0.2% LH and ECI peptides with or without chitobiose units at various concentrations. Experiments under each condition were performed in triplicate wells. The culture media and cells were harvested at 3 days, and used for immunoblotting, enzyme immunoassay, RNA extraction and Northern blot analysis. For Northern blot analysis, cells in triplicate wells were combined to isolate poly(A)+RNA enough for the assay.

Inhibition experiments using anti-emmprin blocking antibody (UM-8D6, Ancell Corporation, Bayport, MN) were performed as described previously [23]. Briefly, ECI peptides with chitobiose units [ECI-(GlcNAc)_2_] were preincubated with the blocking antibody in SF DMEM containing 0.2% LH at 37°C for 45 min and then added to fibroblasts (4 × 10^4^). Their media were replaced once with fresh SF DMEM containing [ECI-(GlcNAc)_2_] and the blocking antibody at 3 days, and the culture media were harvested at 6 days.

### RNA isolation and Northern blot analysis

Poly(A)+RNA was isolated from cultured cells using the Fast Track mRNA isolation kit (Invitrogen, San Diego, CA). Northern blotting was performed as described previously [9]. Cloned cDNA (1,752 bp) encoding human MMP-2 was used as a probe. For internal control of loading, the blots were subsequently hybridized to a glyceraldehyde-3-phosphate dehydrogenase (G3PDH) probe (Clontech, Mountain View, CA). The probes were radiolabeled by random priming with ^32^P-CTP. For quantification of the RNA blot analysis, the radioactivity of mRNA signals for MMP-2 was directly measured by a Bioimaging Analyzer, FUJIX BAS2000 system (Fuji Photo Film, Tokyo, Japan), and normalized relative to the corresponding G3PDH mRNA signals.

### Immunoblotting

SDS-PAGE and immunoblotting of conditioned media were performed using a 5-15% gradient separating gel, Immobilon membrane (Millipore, Bedford, MA) and a mouse mAb to human MMP-2 (75-7F7, Fuji Chemical Industries, Takaoka, Japan) as described previously [23]. To quantitate the relative amounts of the MMPs, the bands on the film were subjected to image analysis (Adobe Photoshop). Statistical analysis was performed using Student's *t*-test.

### Enzyme immunoassay (EIA)

The protein concentrations of pro-MMP-2 were measured using commercially available EIA kit (Daiichi Fine Chemical, Takaoka, Japan) using the protocols supplied by the manufacturer. All assays were performed in triplicate and statistical analyses were performed using Student's *t*-test.

### Zymography

Gelatinolytic activities in conditioned media were demonstrated using gelatin as a substrate. SDS-PAGE was performed under non-reducing conditions using a 9% separating gel containing 1 mg/ml gelatin. After electrophoresis, the gel was shaken gently in detergent buffer (5 mM CaCl_2_, 2.5% Triton X-100, and 50 mM Tris-HCl, pH 7.6) at room temperature for 60 min to remove the SDS, and then incubated in reaction buffer (0.15 M NaCl, 10 mM CaCl_2_, 0.02% NaN_3_, and 50 mM Tris-HCl, pH 7.6) at 37°C for 30 h followed by staining with 2.5% Coomassie brilliant blue in 30% methanol and 10% acetate. Enzyme activity was detected as a clear band on the resulting blue background of undigested gelatin. Gel images were analyzed digitally (Adobe Photoshop) to quantitate relative activities.

## Results

### Effects of ECI with or without a chitobiose unit on MMP production by fibroblasts

**Figure 2:**
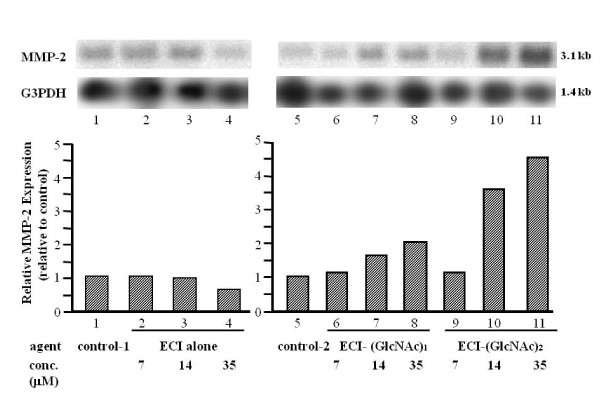
**Effects of ECI-(GlcNAc)_1/2 _on MMP-2 production by fibroblasts - Northern blotting**. After culture of fibroblasts in the presence of synthetic ECI alone or ECI-(GlcNAc)_1/2 _for 3 days, the extracted total RNA was subjected to Northern blotting. *Top panel*, representative blots. *Bottom panel*, MMP-2 expression level relative to the control. Control-1, solvent control for ECI alone; control-2, solvent control for ECI-(GlcNAc)_1 _and _2_.

**Figure 3:**
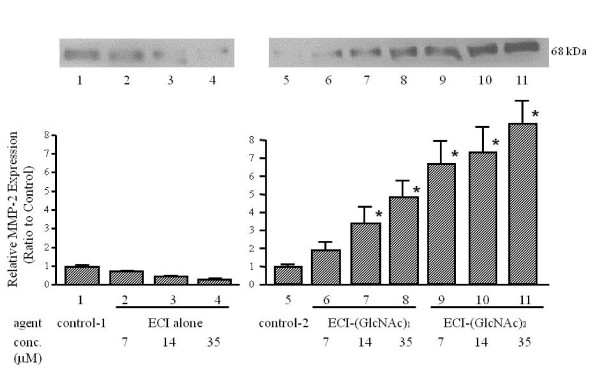
**Effects of ECI-(GlcNAc)_1/2 _on MMP-2 production by fibroblasts - western blotting**. After culture of fibroblasts in the presence of synthetic ECI alone or ECI-(GlcNAc)_1/2 _for 3 days, the conditioned media were subjected to western blotting. *Top panel*, representative blots. *Bottom panel*, MMP-2 expression level relative to the control. Data are mean ± SEM (n = 3). Control-1, solvent control for ECI alone; control-2, solvent control for ECI-(GlcNAc)_1 _and _2_. *p < 0.01, compared with the control.

**Figure 4:**
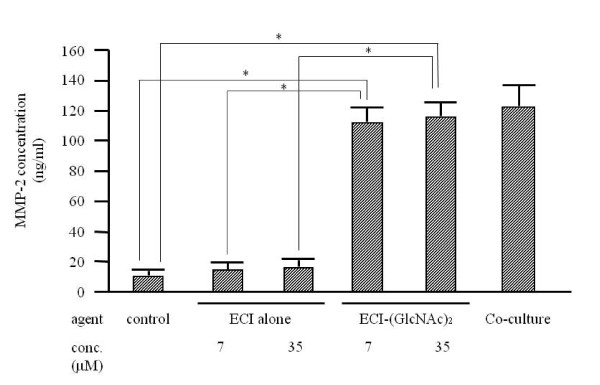
**Effects of ECI-(GlcNAc)_1/2 _on MMP-2 production by fibroblasts - enzyme immunoassay**. After culture of fibroblasts in the presence of synthetic ECI alone or ECI-(GlcNAc)_1/2 _for 3 days, the conditioned media were subjected to enzyme immunoassay. Data are mean ± SEM (n = 3). Control, solvent control for ECI alone and ECI-(GlcNAc)_2_. *p < 0.01, compared with the control.

Previous studies showed two important findings with regard to emmprin activity, namely that the activity resides in ECI [6], and that it depends on N-glycosylation of extracellular domains of emmprin [17,18]. These findings prompted us to synthesize the whole ECI with attached sugar chains (N-glycosylation). In the first step, ECI carrying one or two GlcNAc at Asn^44 ^was synthesized. When added to fibroblast cultures, ECI-(GlcNAc)_1 or 2 _stimulated MMP-2 mRNA expression, while unsubstituted ECI had no stimulatory effect (Figure 2). The stimulatory activity of ECI-(GlcNAc)_2 _was much more than that of ECI-(GlcNAc)_1 _and was dose-dependent: approximately 1.1-, 3.6- and 4.6-fold stimulation at 7, 14 and 35 μM, respectively, compared to control. Stimulation was also demonstrated at the protein level. By immunoblotting, ECI-(GlcNAc)_1 _stimulated MMP-2 production by approximately 2.0-, 3.4- and 4.9-fold at 7, 14 and 35 μM, respectively, compared with the control, and ECI-(GlcNAc)_2 _stimulated it by approximately 6.7-, 7.2- and 8.6-fold, respectively, at the same concentrations (Figure 3). Again, unsubstituted ECI had no stimulatory effect. The stimulated production of MMP-2 protein by ECI-(GlcNAc)_2 _was also confirmed by quantitative EIA (Figure 4). ECI-(GlcNAc)_2 _significantly stimulated MMP-2 production (mean ± SEM, 114 ± 9.8 and 118 ± 8.6 ng/ml) of MMP-2 at 7 and 35 μM respectively, compared to control (9.1 ± 2.4 ng/ml) and ECI alone (14.2 ± 3.3 and 15.2 ± 4.1 ng/ml at 7 and 35 μM, respectively) (*p *< 0.01). The levels of MMP-2 stimulated with ECI-(GlcNAc)_2 _at 7 or 35 μM were similar to those stimulated in tumor cell-fibroblast co-cultures (124 ± 14 ng/ml).

**Figure 5:**
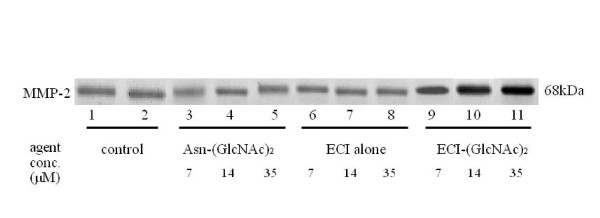
**Effects of the control ECI peptide and the chitobiose unit on MMP-2 production by fibroblasts**. The fibroblasts were cultured in the presence of synthetic ECI alone, Asn-(GlcNAc)_2_, and ECI-(GlcNAc)_2 _for 3 days, and the conditioned media were subjected to western blotting for MMP-2. Control, solvent control for Asn-(GlcNAc)_2_, ECI alone, and ECI-(GlcNAc)_2_.

To rule out that (GlcNAc)_2 _alone stimulated MMP synthesis in fibroblasts, Asn-(GlcNAc)_2 _was added to fibroblast cultures. The results showed no stimulatory effect for Asn-(GlcNAc)_2 _or unsubstituted ECI on MMP-2 production (Figure 5), while ECI-(GlcNAc)_2 _stimulated MMP-2 production.

### MMP-2 stimulation activity of ECI-(GlcNAc)_2 _vs. ECI-[(Man)_3_(GlcNAc)_2_]

**Figure 6:**
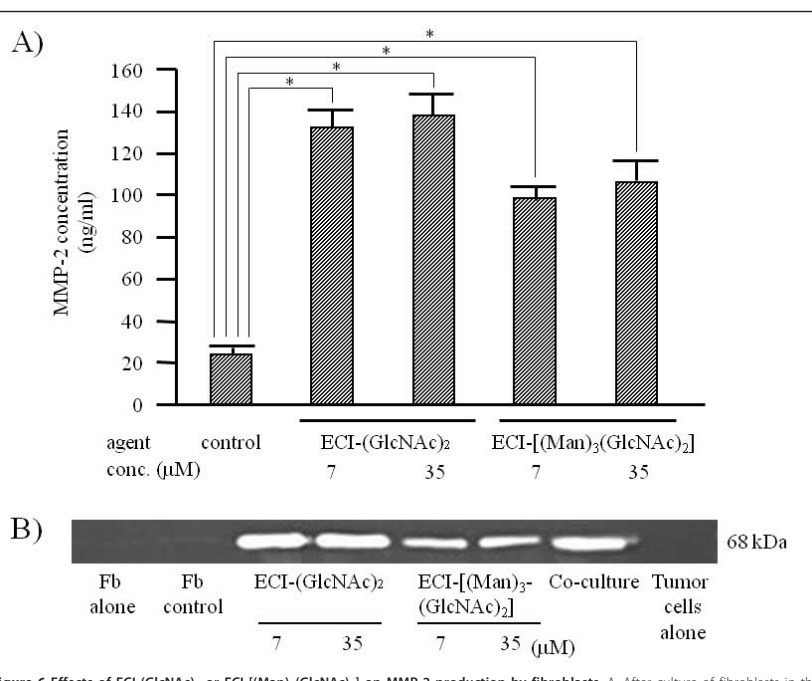
**Effects of ECI-(GlcNAc)_2 _or ECI-[(Man)_3_(GlcNAc)_2_] on MMP-2 production by fibroblasts**. A, After culture of fibroblasts in the presence of synthetic ECI-(GlcNAc)_2 _or ECI-[(Man)_3_(GlcNAc)_2_] for 3 days, the conditioned media were subjected to enzyme immunoassay. Data are mean ± SEM (n = 3). Control, solvent control for ECI-(GlcNAc)_2 _and ECI-[(Man)_3_(GlcNAc)_2_]. *p < 0.01, compared with the control. B, The conditioned media obtained as described above were subjected to gelatin zymography. At the same time, conditioned media from co-culture experiments of FU-EPS-1 tumor cells and fibroblasts were also applied to the same gel. Fb, fibroblasts. Fb control, fibroblast cultures with solvent control for ECI-(GlcNAc)_2 _and ECI-[(Man)_3_(GlcNAc)_2_].

Next, we investigated whether ECI with longer chitobiose units could induce more potent stimulation of fibroblasts to produce larger amounts of MMP-2. Treatment with ECI-[(Man)_3_(GlcNAc)_2_] significantly stimulated fibroblasts to produce MMP-2 (98.7 ± 5.4 and 106 ± 11 ng/ml at 7 and 35 μM, respectively) compared to the control (24.4 ± 3.2 ng/m) (*p *< 0.01) (Figure 6A). However, the extents of stimulation by ECI-[(Man)_3_(GlcNAc)_2_] were lower than those by the same dose of ECI-(GlcNAc)_2 _(132 ± 7.8 and 138 ± 8.5 at 7 and 35 μM, respectively). The extents of stimulation of MMP-2 production by ECI-(GlcNAc)_2 _were almost similar to those by FU-EPS-1 tumor cell surface emmprin in co-cultures of FU-EPS-1 cells and fibroblasts (Figure 6B).

### Anti-emmprin blocking antibody Inhibits the MMP-2 stimulating activity of ECI-(GlcNAc)_2 _peptide

**Figure 7:**
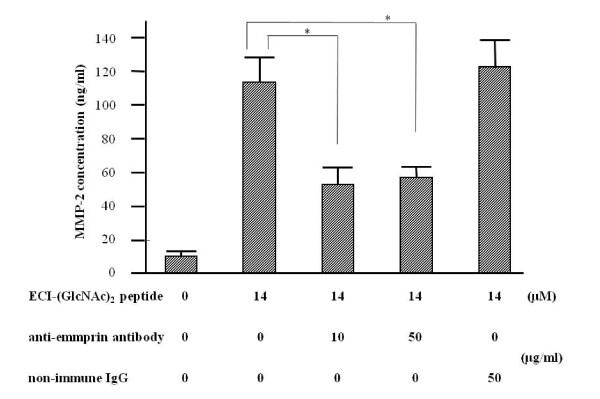
**Effects of anti-emmprin antibody on ECI-(GlcNAc)_2_-induced stimulation of MMP-2 production by fibroblasts**. Fibroblasts were cultured in the presence of ECI-(GlcNAc)_2 _and anti-emmprin antibody or control non-immune IgG for 3 days, and the conditioned media were subjected to enzyme immunoassay for MMP-2. Data are mean ± SEM (n = 4). *p < 0.01, compared with the control.

To confirm that the responses in the above ECI-(GlcNAc)_2 _peptide-induced stimulation of MMP-2 production were emmprin-mediated, inhibition assay using anti-emmprin blocking antibody was performed. EIA showed that the anti-emmprin blocking antibody inhibited (14 μM) ECI-(GlcNAc)_2_-induced stimulation of MMP-2 production (inhibition by approximately 58% at 10 μg/ml and 54% at 50 μg/ml), while non-immune IgG as control did not cause any inhibition (Figure 7).

## Discussion

The present study showed that the synthetic emmprin ECI peptide can exhibit emmprin activity to stimulate fibroblasts to produce MMP-2, when substituted with a chitobiose unit (GlcNAc-GlcNAc). The whole ECI without a chitobiose unit showed no stimulatory activity on MMP-2 production. These findings suggest that ECI is a key domain in determining the activity of the emmprin molecule. Moreover, the results showed that ECI was active, in terms of stimulating MMP-2 production, in the presence of the shortest form of N-glycosylation.

Two groups have reported that emmprin stimulation of MMP production in fibroblasts is dependent on N-glycosylation of its extracellular domains [17,18]. The ability of the emmprin molecule to participate in homophilic adhesion alone is not sufficient for MMP induction because glycosylation is not needed for homophilic adhesion but is essential for MMP-1 and MMP-2 induction [18]. ECI with glycosylation, although just a short chitobiose unit, may send a signal into the cells to upregulate MMP-2. There are two possibilities: First, the carbohydrate side chains may participate directly in ligand binding to a putative fibroblast receptor. Second, they may be necessary for preservation of an active molecular conformation [5]. Intriguingly, in our study, ECI substituted with only two GlcNAcs induced MMP-2 expression at both mRNA and protein levels. This chitobiose unit is the common structure formed at the reducing end of N-linked carbohydrates. Longer and more complex N-linked carbohydrates are reported to be involved in cellular adhesion, possibly via direct binding to lectins [26]. As for emmprin ECI, however, substitution with only two GlcNAcs was more potent than that with the core pentasaccharide. Thus, it is more likely that the chitobiose unit plays a role in preservation of an active molecular conformation. The peptide structures might be bent somehow by hydrogen bonds formed between amino acids and carbohydrates to form an active molecular structure. This possibility is currently under investigation in our laboratories. Similarly, it was shown that the adhesion domain of human CD2 bears a single N-linked carbohydrate, and that the carbohydrate stabilizes the protein fold by counterbalancing the unfavorable clustering of five positive charges centered about lysine-61 of CD2 [27]. Furthermore, the addition of short saccharides (e.g., di-, tri- and tetra-saccharides) to Notch by Fringe is postulated to increase Notch's sensitivity to Delta-like ligands, although they are O-linked carbohydrates [28]. This glycosylation supposedly does not affect Notch signaling by directly influencing the binding between Notch and ligands but may influence steps that occur just before the release of the Notch intracellular domain by proteolytic cleavages [28].

Induction of MMP-1, 2 and 3 mRNAs in uterine fibroblasts by non-glycosylated recombinant emmprin has been reported also [19], although a higher expression at the protein level was not confirmed. The authors hypothesized that their recombinant emmprin maintained the disulfide-stabilized tertiary structures, which let emmprin form dimers in solution and promoted their binding to the cell surface receptors, followed by increased MMP expression. Our study used smaller ECI peptides so that chitobiose substitution might have been necessary to give the peptide some tertiary structures. Moreover, the fibroblasts used in this study were stimulated to produce only MMP-2, but not MMP-1 and 3, in response to tumor cell emmprin (data not shown). Thus, the effect of the ECI peptide with chitobiose substitution on MMP-1 and 3 could not be examined.

Recently, the crystal structure of the extracellular portion of emmprin has been determined [29]. The structure comprises an N-terminal IgC2 domain (corresponding to ECI in this paper) and a C-terminal IgI domain, which are connected by a 5-residue flexible linker. This unique domain organization leads to overall flexibility and diverse dimerization manners of emmprin and may explain its multifunctional character. Another possible explanation for multifunction is glycosylation, since the latter is a highly diverse nontemplate-driven process that can generate enormous informational content [30]. The glycosylated form of emmprin was not analyzed in the above mentioned crystalographic study.

In this study, ECI was shown to stimulate MMP-2 production in the presence of the shortest form of N-glycosylation. On the contrary, we previously reported that ECI-derived peptides inhibit emmprin-enhanced MMP-2 production in the absence of N-glycosylation [11,24,31]. One of the ECI-derived peptides, emp#2, which contains a putative N-glycosylation site but shows no glycosylation, effectively inhibited emmprin-induced stimulation of MMP-2 production. Furthermore, emp#2 peptide effectively inhibited the enhanced invasion of matrigel by glioblastoma cells in the co-presence of fibroblasts [31]. These results also show biological importance of presence of N-glycosylation in ECI.

Thus far, several lines of evidence point to the importance of emmprin in cancer biology. First, emmprin is upregulated in a broad spectrum of malignant tumors, including carcinomas, gliomas and lymphomas, compared with their normal counterparts as mentioned in the Introduction section. Second, emmprin promotes tumor progression as well as upregulation of MMPs in animal models: emmprin cDNA-transfected human breast cancer cells were considerably more tumorigenic and invasive than plasmid-transfected cancer cells when implanted into the nude mouse [32]. Third, as already discussed above, emmprin may be responsible for upregulated MMPs, enhanced angiogenesis and hyaluronic acid production, transformation of stroma fibroblasts, and drug resistance [6,12-18,32]. In this context, it is important to elucidate the mechanisms involved in multifunctionality of the emmprin molecule.

## Conclusions

This study indicates that ECI can mimic emmprin activity in terms of stimulating MMP-2 production by fibroblasts when substituted with chitobiose, the disaccharide with which N-glycosylation starts. This synthetic glyco-peptide may be useful for elucidating the mechanisms involved in glycosylation-mediated emmprin activities, which could lead to new strategies to prevent cancer invasion and progression enhanced by emmprin.

## Competing interests

The authors declare that they have no competing interests.

## Authors' contributions

TK, TS, HH and KK carried out the experiments. KN, HH, BPT, JS and AI participated in the design of the study, its coordination, and helped to draft the manuscript. In addition, technical guidance from YN and YO lead to successful experiments. All authors approved the final manuscript.

## Pre-publication history

The pre-publication history for this paper can be accessed here:

http://www.biomedcentral.com/1471-2407/11/300/prepub
